# Analecta

**Published:** 1842-09-17

**Authors:** 


### ANALECTA.

In the Provincial Medical Journal, June 18, 1842, we find a notice of three cases of empyema, by Mr. Oke, of Southampton.

The first case is as follows:

Case I.—I was requested to visit a lad, the son of a labourer, aged thirteen, suffering with acute pain of the left side, cough, dyspnœa, and fever. His illness had been of short duration, and was supposed to have arisen from cold.

On examining the thorax, I found a swelling a little below, and posterior to, the left nipple. The swelling was fluctuating and without pulsation, and indicated the presence of pus in large quantity ; nothing, in short, could be more easy than the diagnosis in this case.

A free incision was immediately made, and a torrent of healthy pus poured from the wound. The quantity measured ninety-six ounces, and must have filled the whole of the left thoracic cavity. The lad was greatly relieved by the evacuation, and in a few weeks he completely recovered.

This is a rare case of cure after operation. The patient was placed under the most favourable circumstances—young and evidently free from tuberculous disease. At the same time, he was old enough to take the necessary care of himself after the operation. In cases like this, we conceive that the operation is justifiable, provided the quantity of pus be so great as seriously to impede the respiration ; otherwise it is at least unnecessary, for absorption generally takes place very rapidly in young subjects so soon as the inflammation is relieved.

In the second case, that of a man forty years of age, there was complete flatness in the whole left side of the chest, without distension. The respiration was absent, and the heart thrust to the right side of the sternum. A mercurial treatment produced absorption, as it almost always does in these cases. A blister, or rather a succession of blisters, generally hasten recovery ; they were not, however, used in this case.

In the third case paracentesis was performed.

Case III.—Robert Gee, aged 33, steward of a steam-vessel, dark hair, pale complexion, and considerably atrophied, was admitted into the South Hants Infirmary on the 8th of March, in the present year.

The following is the history of his case :—

He has been affected with cough since February, 1841, which originated with symptoms of common nasal and bronchial catarrh. As the attack was not severe, he thought nothing of it, and continued his duties on board the vessel. The cough, however, did not leave him ; and after about four months he began to feel pain of the left side, the muscles of which became stiff, and interrupted the free use of his left arm. These symptoms went on more or less till October, when the pain of the side increased to such a degree, that he felt as if knives were piercing him ; at the same time the cough also increased, associated with dyspnœa and muco-purulent expectoration.

When I visited him on the 14th of December, these symptoms had considerably subsided. There remained a troublesome cough, with scanty expectoration, and a sense of weight of the left side, on which alone he could lie with any degree of comfort. There was some degree of attenuation ; the pulse ranged from 90 to 100 in a minute ; the tongue was clean, and his general health not much disturbed. On exploring the thorax, the left side was dull by percussion, and but very limited respiration could be detected by auscultation, and that over the upper lobe ; on the right side, the percussive sounds were clear and loud, and the respiration was puerile.

As he had been the subject of cough so long, and was one of a consumptive family, I supposed the case might consist of disorganization of the left lung, and, therefore, did not expect that it could admit of any essential service being done ; however, I placed him under a mercurial course in the following formula :—

Calomel, one grain ;
Extract of conium, three grains. A pill, thrice a-day.

By this means he was smartly ptyalised for several weeks; but he did not appear to be benefitted by the treatment, either locally or generally; on the contrary, at the expiration of two months he had lost flesh; his breathing had become more laborious; the left side was everywhere dull; its external aspect was enlarged, measuring transversely an inch more than the right; the intercostal spaces were dilated, and without muscular action; no respiratory sound could be detected at any part of the left side; and the heart was pushed to the right side of the sternum. Along with these symptoms, there was serous infiltration into the sub-cutaneous tissue over the inferior part of the left side of the thorax, and into the peritoneal cavity; there was also anasarca in a slight degree of both inferior extremities. What is somewhat remarkable, the cough and expectoration had almost subsided.

On the 9th of March, the day after his admission, a grooved exploration-needle having discovered the presence of pus, a free incision was made by a scalpel, through the sixth intercostal space into the chest, midway between the ensiform cartilage and the tenth dorsal vertebra. A mass of coagulated albumen plugged up the aperture; but as soon as this was removed, ninety ounces, or five pints, of laudable pus were evacuated.

He bore the operation well, and was greatly relieved by it. For some days the same albuminous mass continued to impede the discharge, which somewhat oppressed his breathing; a free and regular outlet was, therefore, subsequently maintained by the frequent use of a director.

The next symptom that presented itself to my notice was the metallic tinkling, accompanied by occasional and forcible expulsions of air through the external aperture. The tinkling was heard through the stethoscope, at any part of the left side of the chest, either after speaking or coughing. It sometimes closely imitated the striking of a wire against an empty glass vessel; at others, the falling of a drop into a thin fluid. Whilst auscultating the actions of the respiratory function a few days ago, I could distinctly hear the air rushing through the pleura pulmonalis into the thoracic cavity; and the sound exactly imitated the blowing of air by the mouth into a large bladder.

The fluid at present discharged from the external orifice is partly serous and partly muco-purulent, the latter kind sticking to the vessel, as in catarrhus vesicæ.

The cough and expectoration returned after the evacuation of the abscess, and have continued more or less to this time; the urine is high-coloured and scanty; there still remains some serous fluid in the cavity of the belly, and the legs are slightly anasarcous above the ancles.

On the other hand, the breathing continues much relieved; there is no hectic development; the pulse ranges from 90 to 100 in a minute; his nights are tranquil, and he can sleep on his back; his appetite is good; he has gained flesh, and can walk to a considerable distance without fatigue.

The percussive sounds are much clearer, and limited respiration can be detected in both lobes of the left lung; the heart and ribs have resumed their normal position; the intercostal muscles are in perceptible action, and the metallic tinkling is seldom heard.

[The issue of this case is not given, but there is little doubt that the termination will not be in perfect recovery. Most of those cases in which the operation is performed terminate in hectic, or at least end in phthisis; which is by no means an uncommon result of empyema, even if the operation be not performed. But the exposure of a large cavity to the air, which can never be entirely avoided, is, of itself, a cause of intense irritation, and one sufficient to prove fatal. As a necessary evil, when there is danger of suffocation from the pressure on the pus, on the mediastinum, and consequently on the healthy lung, we admit the operation of empyema; but in most cases, the treatment by mercurials and repeated blisters is so effectual, that an operation is, to say the least of it, unnecessary. When the pleurisy is complicated with phthisis, the prognosis is, of course, unfavourable. W. W. G.]

---

*Professional Incomes in London.*—An interesting Memoir of the late Dr. James Hope, the distinguished author of the Treatise on Diseases of the Heart, has been recently published by Mrs. Hope. The following particulars, in relation to the incomes of the first grade of practitioners in London, will be read with interest:—

On arriving in London, Dr. Hope was led into the belief that the first twenty physicians of the metropolis divided about £80,000 annually between them, and that a successful physician might hope to be established in good practice in about five years. To be one of so large a number as twenty seemed no difficult task, and therefore "he ignorantly hoped" that if he succeeded at all, he should be making £4000 a year in about five years! He was, of course, quickly undeceived. He had now gained many friends, he was married, he was a successful author, but the approaches to £4000 a year were "very tardy." He thought himself in fault, until a little friendly conversation with Dr. Chambers and Sir Henry Halford taught him more correctly upon the subject of so speedily stepping into large receipts. Dr. Hope kept a regular account of every fee which he received during the twelve years he was in London. "We are, therefore," says the Editor, "enabled to speak with the greatest accuracy." The first two years he was in London he made £200 a year. The third year, the accidental removal of some families who employed him, reduced his practice to £150. At the end of the third year his work on the Diseases of the Heart was published, and he came before the profession as physician to the Marylebone Infirmary. Still, in the first year after, his practice was little increased; but from this period his reputation became extended, and his practice gradually increased, until in eight years more, when he retired, he was making £4000 a year. This—as we should call it—great success was fairly and honourably laboured for and won. —*Lond. Med. Gaz.* July 29, 1842.

---

*M. Bourgery on the Anatomy of the Spleen.*—M. Bourgery considers the spleen to consist of ten component parts:—1. Vesicular membranes; 2. blood-vessels; 3. floating vascular corpuscules; 4. a granulo-capillary ground; 5. a splenic liquid; 6. splenic glands; 7. lymphatic vessels; 8. nerves; 9. cellular tissue; 10. the enveloping membrane of the spleen.

The splenic vesicles are uniformly spread over the whole extent of the organ, are of an irregular polyhedral, and, when fully injected, of a spheroidal or oval shape, varying in size in different animals, and also in the same spleen. In man their mean diamater is from 1 to $1\frac{1}{4}$ millimetre. Each vesicle does not form a simple cavity, its walls being traversed by vessels, which form projections in the interior, covered by the lining membrane. In consequence of this arrangement the cavity is divided into cells, at the bottom of which the minute glands and capillary vessels are seen in relief. There are orifices in vesicles of two sorts:—1. Orifices of communication more or less irregularly circular, whose edges are thin, and formed by a fold of the membrane which lines the cavity. Their diameter is from a quarter to half of that of the vesicle. There are one, two, or three orifices in each vesicle; and to this reciprocal communication is due the ready inflation of the organ, not only from the veins, but also from an aperture on any part of its surface. 2. The venous orifices, less numerous than the communicating, and scattered here and there, a single vesicle sometimes containing two or three, at others, one cannot be found in a group of several vesicles. They are of a circular or elliptical shape, one-twelfth of a millimetre in diameter, and are the absorbing mouths of veins. Between the vesicles are septa, or intervesicular spaces, formed by the separation of their lining membrane, and containing the splenic glands and vessels, their size varying with the greater or less repletion of the organ, but generally bearing the relation of 2 to 3 to the vesicular part. The investing membrane, forming the walls of the cells, is continuous through the whole extent of the spleen, and may be considered as one homogeneous whole, divided into numerous little ampullæ. It is supported by vessels and glands, and forms a sort of frame-work to the organ. This membrane is very complicated in its structure, and cannot therefore be considered, as by Malpighi, to be a simple dilatation of the internal tunic of the veins.

The splenic arteries and veins, running side by side, are directed towards the periphery of the spleen; the veins being pierced in their whole extent by little circular apertures, leading into the smaller intervesicular branches, which are distributed to the splenic glands and the membranes of the vesicles. The small vessels project into the cavities of the vesicles in a peculiar manner to reach the floating corpuscules, and present the appearance of a bunch of grapes. Lastly, all the small vessels of the spleen, in the turgid state, present numerous dilatations and contractions, so as to give them a marked knotted appearance.

The corpuscules are small bodies floating in the cavities of the vesicles, to the walls of which they are attached in a pediculated manner, by the extreme branches of the blood-vessels and lymphatics. They are about fourteen or fifteen times the size of the blood-globules. By the granulo-capillary basis is intended to be represented the two structures placed beneath the vesicular membrane, viz. spherical pale granules, or glands, four or five times the diameter of the blood-globules, and the capillary net-work of veins, arteries, and lymphatics.

The splenic liquid, which appears to be elaborated by the floating corpuscules and the granulo-capillary basis, is deposited in the cavities of the vesicles, or taken up by the absorbing veins or their walls. It is thick, viscous, of a reddish-brown colour, and under the microscope appears to consist of globules suspended in a yellowish unctuous fluid. 1. Lenticular globules, some of which are surrounded with a red border, and appear to differ very little from ordinary blood-globules; others being colourless. 2. Whitish globules, irregular in form and size, and resembling those found in the chyle and lymph.

The splenic glands, so far as actual volume and consistence are concerned, form the principal organic element of the spleen. They fill, with the ramifications of the vessels, the intervesicular spaces. Their diameter is a quarter of a millimeter. They are united by bands of a similar nature with themselves, which extend over every part of the spleen. From the afferent and efferent lymphatic trunks, and the dispositions of the vessels about them, M. Bourgery is convinced that they are simply minute lymphatic glands. The lymphatic vessels are extremely numerous in the spleen, and in many situations remarkable in their structure, being enlarged at intervals, and, in addition to their valves, divided in their interior by septa, which gives them a sort of rudimentary glandular structure, and shows them to be not merely canals for transport, but also, in some degree, organs of elaboration.

M. Bourgery considers the spleen as an apparatus for the elaboration of the blood, and draws an analogy between it and the lymphatic glands; observing that if, in relation to its anatomical structure, we may define the spleen as a vast lymphatico-sanguineous gland, on the other hand, lymphatic glands generally may be considered, to a certain extent, as small spleens appended to different parts of the circulatory apparatus. In treating of the intimate structure of these glands, we shall see how far the idea of the conformity of these two sorts of organs, evident so far as the glandular structure of the spleen is concerned, is borne out by the internal organization of the canals of the lymphatic glands.—*Ibid, from Gazette Médicale.*

---

*Case of Excision of the Upper Maxillary Bone.* By R. D. Mussey, M. D., Professor of Surgery in the Medical College of Ohio, and Surgeon of the Commercial Hospital at Cincinnati.—Thomas McGillighan, a locksmith, æt. 22, consulted me, in July, 1839, for a painful affection of the left side of the face, which had existed about eight months. The left nostril was entirely blocked up by an adventitious growth of considerable firmness, which extended anteriorly within half an inch of the margin of the ala and septum, and posteriorly so far as to be felt by the finger above the floating edge of the soft palate. The ceiling of the mouth, on the left side was pushed downwards, so as to present a slight convexity, and the cheek was more prominent than the other. For the pain, which extended along the alveolar arch, he had several teeth extracted, but without any important relief. The general health was not materially affected. As there could be no doubt that the tumor sprung from the antrum, and as its progress had been somewhat rapid, I recommended the excision of the jaw-bone as soon as the hot weather should subside, and a strict adherence to a farinaceous diet, with water and a small quantity of milk for drink, which course was faithfully pursued.

On the 28th of September, 1839 I performed the operation in the following manner:—An incision through the integuments, commencing a quarter of an inch below the tendon of the orbicularis palpebrarum, was carried down by the side of the nose, and close to the convex border of the ala, thence horizontally to the median line, from which point the upper lip was cut through vertically. Another curvilinear incision extended from the angle of the mouth to the outer margin of the bony orbit as high as the external canthus. The flap included between these incisions was dissected up and thrown upon the forehead, and the malar bone was exposed by a horizontal incision of an inch backward along the zygoma from the margin of the second incision. An incision on the median line from the incisors to the posterior edge of the hard palate, through the lining of the arch of the mouth, and another through that of the palate, separating it from the palate plate of the palate bone, completed the section of the soft parts. By the aid of a saw and bone nippers, the bony connections were divided, and the whole of the upper maxillary bone, except the point of its nasal process—which was left on account of the lachrymal sac—was removed, together with a part of the malar, and the whole of the palate plate of the palate bone. The tumor occupied the cavity of the antrum, had pushed through its anterior wall, and attenuated its flooring, filled up the whole nasal avenue, pressed the septum some way into the right nostril, and crowded itself into the cells of the sphenoid bone, and, if I judge correctly, filled up the whole cavity of the body of that bone. From this situation I dug it out with the point of my finger.

There was not much hæmorrhage. Three or four vessels only required the ligature. The flaps were preserved in situ by stitches, and a great part of the wound united by adhesion. No severe pain nor considerable constitutional irritation followed the operation, and on the *tenth day* the patient took a walk in the street. The tumor was firm and somewhat fibrous in some parts, and decidedly encephaloid in others. From its soft and homogeneous texture, I entertained fears that it might return, and enjoined it upon the patient to live without flesh, fish, or greasy food, with no condiment except salt, and to drink nothing but water—a course which he has rigidly followed to the present time. He has enjoyed fine health, without a trace of the disease, since the operation—a period of *two years and nine months*. The winter after the operation, Dr. Cook, an ingenious dentist of our city, inserted a gold palate, with an arch of teeth, which restored a natural appearance to the mouth and a perfect articulation. This is still worn, and so slight a deviation from symmetry between the two sides of the face exists, that very few would suspect any operation to have been performed upon it.

Mr. McGillighan is now a thriving mechanic, and a worthy citizen.—*Western Lancet.* August, 1842.

---

*Ligature of the External Iliac Artery for Femoral Aneurism.* By Wm. Power, M. D., one of the Attending Physicians to the Baltimore Almshouse Hospital.—Christopher Daly, a native of Baltimore County, æt. 61, entered the surgical ward on the 1st of July; a man of stout frame, very youthful appearance for one of his years, and enjoying excellent health with the exception of the aneurismal disease for which he came to the hospital. For the last fourteen years he has enjoyed good health, uninterrupted by an attack of any sort unfitting him for his daily labour. About fifteen months ago, according to his best recollection, his attention was first accidentally called to the aneurismal tumor whilst digging in a ditch, and standing in water, though with his feet well protected. Placing his fingers upon it, he says he "distinctly felt a pulse, but thought it was in a very strange place."—As it gave him no pain or inconvenience he thought it was nothing serious and continued to work on as usual. About a month had elapsed from this time, when he was suddenly seized with violent cramps in the diseased limb, and finding exertion painful, he stopped his usual employment, but assisted during the following harvest in the lighter kinds of field labour. The limb was slightly swollen below the tumor when he first discovered it; but after the attacks of cramp, which were from time to time repeated, the swelling increased, together with a sense of numbness diffusing itself over the whole leg. The tumor has steadily continued to increase, together with the œdema of the leg. He has, for the last eleven months, kept quiet and strictly avoided all exertion, and has undergone no treatment until he came under our care. The aneurismal tumor was situated in the right groin immediately below Poupart's ligament, which was forced a little upwards in an arched form. It was of an irregularly rounded form, about three and a half inches in its perpendicular, and three in its transverse diameter—pointing a little upwards and inwards, where the pulsation was more distinctly seen and felt than elsewhere. Its pulsation was evident to the eye—communicated a distinct thrill to the hand when laid upon it, a *bruit de rape* to the stethoscope, and the pulsation were eccentric. Compression upon the iliac artery, on the psoæ muscles, arrested all pulsation in the tumor. The external iliac for an inch above the tumor felt larger than the artery of the opposite side, and both here and in the continuation of the femoral below the tumor, gave a distinct file-sound to the stethoscope. The lymphatic glands of the groin were swollen and hard—the leg was one-third larger than that of the opposite side—purplish in hue, one degree higher in temperature, affected frequently with cramps, and pain with a sense of numbness over the knee and instep.

Tongue clean, appetite good, bowels regular—skin pleasant, pulse 80, natural, sounds of the heart clear and distinct, chest every where resonant, respiratory murmur vesicular, no ossification of the arteries at the wrist. The rest and quiet of the hospital reduced in a degree the œdema of the leg, and caused the tumor to appear more prominent. He was placed on mild diet, and one or two gentle aperients administered. His health remained undisturbed, and his spirits good up to the 19th of July, when, assisted by my colleague, Dr. Annan, and in the presence of several medical friends, the following operation was performed.

An enema having been previous administered, and gutt. lx. Tr. opii, given an hour previously, the patient was placed upon a suitable table, his pelvis inclining towards the left side, and an incision made through the skin and common integuments of three inches and a half in length, extending from three-fourths of an inch to the inside, and on a level with the antero-superior spinous process of the ilium, with a curve presenting downwards to about three-fourths of an inch above the external ring—a small vessel running to the skin was divided in the inner angle of this wound and secured. The glistening fibres of the fascia of the external oblique were then divided, according to a suggestion in the last edition of Dr. N. R. Smith's work on the Arteries, in the same direction, but a half inch above Poupart's ligament. I now attempted to pass my finger under the edge of the external oblique and fascia transversalis, but found the border of the fascia of the external oblique presented such obstruction to free manipulation, by its resistance, that, to facilitate my farther progress, I was obliged to divide it with a buttoned bistoury, thus making a T incision in this fascia. The finger now passed readily down on the outer side of the internal ring, avoiding the cord, and after a little dissection with the nail, came in contact with the artery. Fearing, however, the soundness of the coats of the vessel thus low down, the sac of the peritoneum was gently pushed upwards and separated from the fascia transversalis for an inch and a half; and this membrane, together with the fibres of the internal oblique and transversalis divided by a buttoned bistoury on the finger for three-fourths of an inch. This sufficiently enlarged the wound for easy manipulation. The finger sought the artery about three inches from Poupart's ligament, and with one of Prof. Gibson's needles, arme t ha ligature of two strans of saddler's silk, waxed but not twisted, an attempt was made to pass it from without inwards ; finding the curve of the needle so great that I could not depress the handle sufficiently on the abdomen, I cautiously separated with the nail the vein from the artery, and passed it from within outwards. The ligature was then drawn tight with some difficulty at this depth, my assistant pressing on the knot until the second was tied. The pulsation immediately ceased in the tumor. The wound was brought together by two points of suture and a few adhesive straps. The operation lasted 25 minutes, and was borne extremely well by the patient. The patient after the wound was dressed was carried into a well ventilated room, and placed on a bed with the limb of the side operated upon well flexed on the pelvis.

Complains only of fatigue and numbness of the right limb, with dull pain of the loins and hypogastric region—pulse 80—natural—at 7, P. M. Temperature of right foot, as tested by the thermometer, seven degrees lower than that of the opposite side—gave Tr. opii, gutt. xxx.

July 20th. Patient rested tolerably during the night—slept nearly four hours ; countenance cheerful ; some nausea and anorexia. Pulse 80, as yesterday ; skin pleasant ; complains of dull pain in the loins, and some cramps in the leg. Tumor decreased one-third in size ; hard on its external side ; slight pulsation at its inner and upper portion. Right leg florid, only two degrees lower in temperature than the left ; ordered venesec. 14 oz. ℞. Morphiæ acet. gr. ij.; aquæ camphor, ℥iv.—dessert-spoonful every two hours. Towards evening his pulse rose to 114. Takes barley water made cold with ice.

July 21st. Appearance much altered ; countenance anxious, haggard ; eyes sunken and suffused ; voice weak ; nausea, constant retching ; complete anorexia. Tongue reddish along the edges ; thirst ; skin bathed in perspiration ; pulse 140, small ; abdomen tympanitic ; complains of sharp pain along the recti muscles, in the iliac region and about the loins ; bladder irritable, trying to make water every ten minutes ; venesec. oz. 14. ℞. Calomel grs. xv., pulv. opii, grs. ij. Added two drops of hydrocyanic acid to each dose of the solution of morphine ; forty good leeches applied to the abdomen. At 7 P. M. the leech-bites were still running freely, and had afforded great relief ; pulse 130 ; blood drawn this morning well buffed ; still restless. Continue anodyne. Tumour as yesterday. Temperature equal on both sides.

22d. Countenance much improved ; pain and uneasiness much less ; abdomen still tympanitic ; no movement of the bowels. Pulse 125, fuller but soft ; tongue moist ; thirst less, and asks for food. Still complains of pain in the back and abdomen ; bladder still irritable ; venesec. 8 oz. ℞. Cal. and opium as yesterday ; hop fomentation over abdomen. 7 P. M. Still improving ; skin pleasant ; demands food ; no passage ; blood drawn in the morning lightly buffed ; the bleeding made a sensible impression upon his pulse. ℞. Cal. grs. viij.; opii, gr. j.; chicken water, and milk iced. Continue solution.

23d. Passed a good night, and makes no complaint this morning excepting of his bladder ; abdomen much softer ; skin pleasant. Pulse 96, soft ; tongue clean ; no stool ; little thirst ; wound gives no pain ; an erythematous blush surrounding it for some inches ; complains none of the limb. ℞. Sal Rochelle, ℥j.; aqua, ℥viij.; wine-glassful every hour. 7 P. M. Bowels not moved ; complains of flatus. Ordered a simple enema.

24th. Looks as well as before the operation ; makes no complaint ; pulse

90, soft; tongue clean. Peritonitis fairly subdued, and we looked forward to a happy result; waking from a sleep at one o'clock he turned over in bed; his nurse a few minutes after was struck by his respiration and altered facies, and a quantity of blood in the bed. I saw him a few moments after, pale, restless, pulse scarcely perceptible, almost unconscious, his bed floating in blood—between two and three quarts. The hemorrhage had ceased; a compress was laid over it; nor did it return; he sunk rapidly, and died about 7 P. M. It was not deemed advisable to make any effort to secure the vessel, as the slightest shock would have determined dissolution under our hands.

*Autopsy eighteen hours after Death.*

Weather very warm. Head and face purplish; features swollen; bloody serum exuding from eyes; slight greenness about the clavicles and abdomen; rigidity considerable; muscles large and firm. The lips of the wound were united, excepting for a half inch on either side of the ligature; this space occupied by a clot of blood; the points of suture had held firmly, and there was no disturbance of parts.

The abdomen was opened by incisions from the umbilicus to the pubis, and to the edge of the ribs. Their flaps turned down exposed the peritoneum. There was no effusion of serum; no adhesions; but over the tract of the artery, extending upwards towards the kidneys, and down into the pelvis to where the peritoneum invests the bladder, this membrane, in a space equal to the size of the hand, was of a bluish livid tint, more wrinkled than elsewhere, as if lightly parboiled, presenting numerous whitish yellow points, which could be scraped off with the nail; some flakes as large as a half-dime. The membrane was firm; no adhesion with the intestine. The peritonitis had been local, and was entirely subdued. The adhesions of the lips of the wound were then torn open; the skin and cellular tissue were the only parts adherent. The blood confined in the wound had insinuated itself upwards for some distance between the fascia transversalis and the peritoneum, as well as between this membrane and the fascia iliaca; about four ounces of clotted blood were removed from the wound. The artery was then exposed, and found with the ligature around it; when pressed upon, blood came from an opening immediately under the knot. It was then carefully dissected out and removed, from Poupart's ligament to the bifurcation of the aorta, and laid open, as well as the external iliac of the opposite side. They were equal in size; at least one-third of an inch in diameter; the inner coat of a creamy yellow hue; a little rough. The inner coat, where the ligature had been applied, on the posterior half of the artery, was wrinkled and crimped, so as to be smoothed out with some difficulty, but was not cut through, nor was there any effusion of lymph. On the anterior part was the opening, at least one-half the circumference of the vessel. The edges a little ragged, of a purplish hue, within a quarter of an inch of the opening; friable, and breaking easily under the nail. The outer coat was livid to the same extent; softened and more friable than the rest of the vessel; a soft clot below, but none above the ligature. The vein perfectly sound; no further separated from the artery than by the needle in passing the ligature; this was applied about an inch and a half below the bifurcation of the common iliac, and about the same distance from the origin of the epigastric. The tumour in the groin was next laid open and cleansed of its clots. It was about three inches in each diameter, the anterior wall formed by the fascia lata, its lateral and posterior walls by the muscles of the thigh; irregularly anfractuous. The profunda, which was given off very high up, immediately under Poupart's ligament, formed a portion of its posterior boundary. The artery, for an inch above where the tumour commenced, was filled with osseous concretions, grating under the scalpel, and pricking the fingers. The continuation of the superficial femoral where it emerged from the tumour, from the number and regularity of the circular atheromatous bands, looked more like the trachea than an artery. The aneurismal tumour was very thin and prominent towards the upper and inner part of the thigh. It contained between four and six ounces of soft black clotted blood, and as much firm fibrin arranged in layers penetrating into the anfractuosities of the tumour.

The arterial system was everywhere well developed; these vessels having more than their usual calibre. The heart was large and flabby, its walls thin, a little fatty; the valves all sound; aorta not dilated; other viscera of the chest and abdomen healthy. Head not examined.—*Maryland Med. and Surg. Journal*. September, 1842.

---

*Tendency of Bad Living to produce Chorea*. By Dr. W. D. Chowne.—Chorea is frequently attributed, and with great justice, to bad and insufficient diet; that diet not sufficiently nutritious may, by producing debility, lay the foundation for chorea, we may be assured; but it is necessary not to lose sight of another fact, that good and sufficient diet may in a defective state of the health, fail to afford good and sufficient nourishment; for it is not enough that proper food should be taken into the stomach, but it is necessary also that it should be properly dealt with when there, lest it should be converted into what is not merely without elements of a nutrient quality, but lest it should be converted into elements that are noxious. It is scarcely possible in practice, whether with regard to children or with regard to adults, to give this fact too much consideration. We perceive that all the three patients to whom I have directed your attention had good and sufficient food, including meat once every day. In one instance (the girl) the appetite was good, and there was no sensations of indigestion until just prior to her coming into the hospital. In another (Godfrey) the appetite was delicate, but the digestion, so far as the patient's sensations were concerned seemed not to be good. A third patient (Watson) had a good appetite, and he did not have any sensations, leading him to consider that his digestion was defective; yet in all these the dejections, when active purgatives were given, proved to be unnatural; and one patient, the girl, says, that although her appetite was good, and before she had any feelings of bad digestion, yet even under these seemingly satisfactory signs she had observed that what she took passed undigested. We have in this particular, demonstration in our three little patients of that which so often happens, and lulls persons into false security, assuming that the vital functions are all going on healthy, because there is no striking, flagrant, self-evident demonstration of the contrary.—*London Lancet.*

